# Precocious Cone Formation Observed on a Three‐Year‐Old Giant Sequoia Seedling

**DOI:** 10.1002/ece3.72621

**Published:** 2025-12-17

**Authors:** Emily V. Moran, Diane Huebner

**Affiliations:** ^1^ Life & Environmental Sciences Department University of California Merced California USA

**Keywords:** fecundity, giant sequoia, life history, reproduction, seed cone

## Abstract

There is thought to be a tradeoff in forest trees between growth potential and reproduction, particularly at early life stages, which leads to a positive relationship between maximum tree size and mean age or size at reproduction. However, as with many traits, variation in reproductive timing can exist within species. Here, we report a case of precocious female cone formation in a three‐year‐old giant sequoia seedling (*Sequoiadendron giganteum)* observed at a research plot in Sequoia National Park, California, USA. Given that sequoia groves are threatened by increasing frequency of high severity fire, this observation suggests that a more systematic study of size/age of earliest reproduction in sequoia is warranted, as reproduction of small trees could affect the likelihood of population persistence.

## Graphical abstract

Here, we report a case of precocious female cone formation in a three‐year‐old giant sequoia seedling (*Sequoiadendron giganteum*). Given that sequoia groves are threatened by increasing frequency of high severity fire, this observation suggests that a more systematic study of the size/age of earliest reproduction in sequoia is warranted.

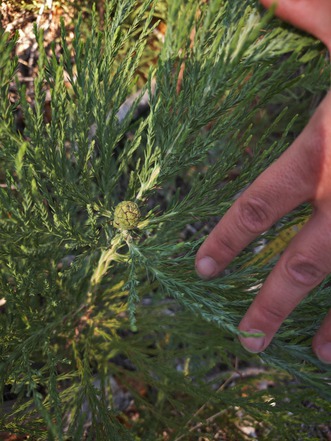

## 1 Introduction

Due to a hypothesized tradeoff between investment in current and future reproduction, it has long been thought that larger trees would tend to mature at greater sizes (Stearns 1989). A recent analysis of 486 species from across the world confirmed that this is the case, though the minimum size at reproduction is proportionally smaller for large species (Journé et al. 2024). Investing in reproductive structures early can slow a tree's growth, with potential costs for survival or later reproduction (Kozlowski 1992). Journé et al. (2024) found that the diameter‐at‐maturation estimate for gymnosperms ranges between 10 and 100 cm. Because giant sequoias (*Sequoiadendron giganteum*) are not only the largest tree in the world by volume, but a species that is vulnerable to the historically‐common ground fires needed for its seedling establishment until sufficient height and bark thickness has been achieved (Lanner 2002; Weatherspoon 1990), one would expect them to delay reproduction until they are past the “fire safe” size. For moderate/prescribed burns, this threshold size is around 30 cm in diameter (Lambert and Stohlgren 1988). However, there may be much more individual variation in size at maturation in this species, and perhaps other trees, than has been previously recognized.

**FIGURE 1:**
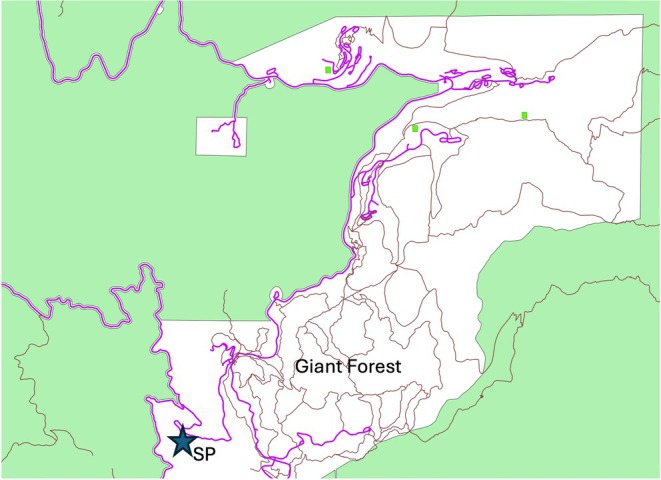
Location of site within Sequoia National Park. Green areas are designated as wilderness. Small green squares are the other long‐term field sites maintained by Moran lab.

While carrying out a routine seedling transect survey in June 2025 at a field site in Sequoia National Park (California, USA), located just below Giant Forest (Figure 1), we observed a giant sequoia seedling that had produced a female cone. This was surprising to us, as such early reproduction had not, to our knowledge, been observed in most conifers, let alone large, slow‐maturing species such as giant sequoia. The seedling in question was 65 cm tall and could not have been more than 3 years old because, in late 2021, the 88,307‐acre KNP Complex Fire caused the area to burn at high severity, killing all seedlings and most canopy trees within the mapped stand. However, a band of trees near the road survived, including two mature giant sequoias (*Sequoiadendron giganteum*) outside of the mapped stand, and seedling recruitment, particularly of sequoias, was high in the subsequent 2 years. Here we report the details of this observation and discuss its potential implications.

## 2 Materials and Methods

The field site in question, known as “SP” (Latitude 36.552981, longitude −118.7830499, elevation 1806 m), was established in 2015 for the purpose of forest demographic measurements and as a location for a seedling planting experiment. Based on charcoal present in the area, the location had experienced an understory fire some time prior to 2014. As per National Park instructions, the research site was placed so that the two mature sequoia trees were outside of its boundaries, so as to minimize trampling around their roots.

**FIGURE 2:**
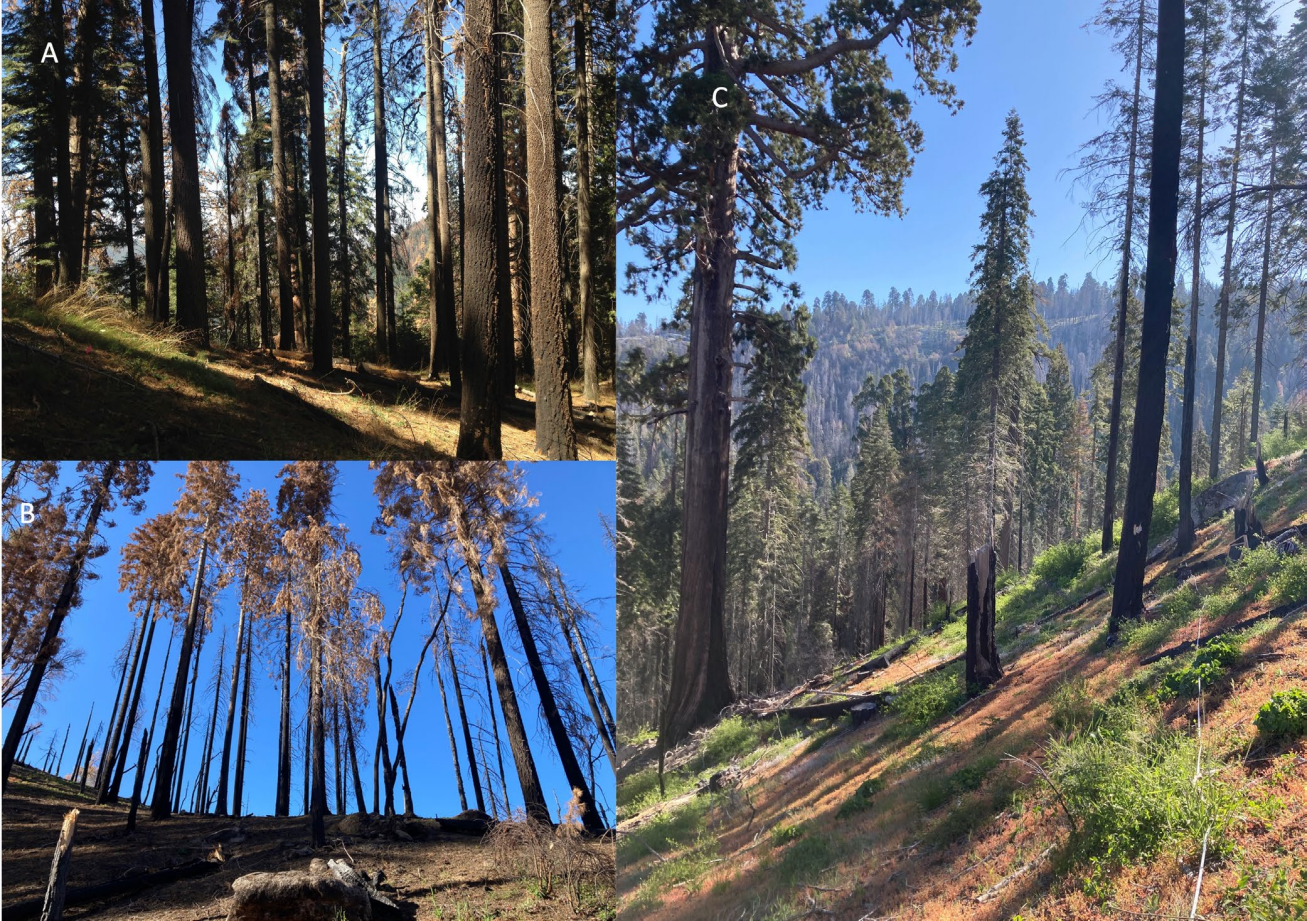
(A) Site in 2016, with dense overstory of fir and pine. (B) Site after KNP fire, 2022. (C) View from interior of site toward adult sequoias and other living trees at road border and beyond. Notice charred trunks in foreground and transect tape to the right. (Photos by Emily Moran).

**FIGURE 3:**
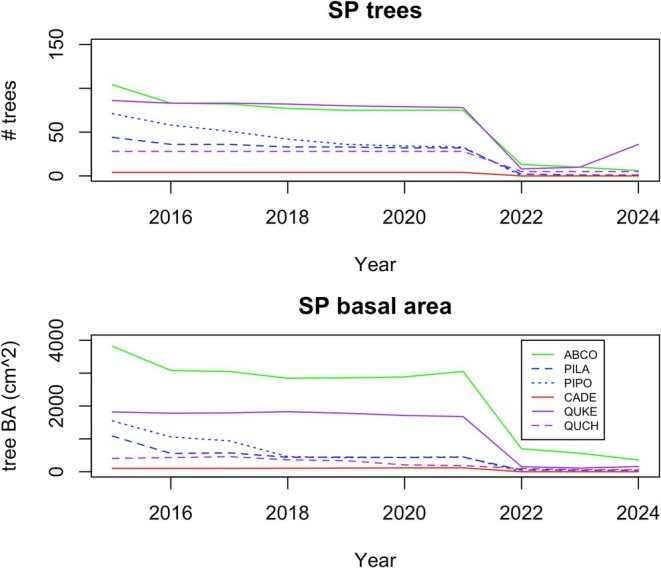
Tree number and basal area over time. ABCO = *Abies concolor* ; QUKE = Quercus kellogii; PIPO = *Pinus ponderosa* ; PILA *Pinus lambertiana* ; QUCH = *Quercus chrysolepis* ; CADE = *Calocedrus decurrens* .

The dominant species of adult tree within the 0.8 ha plot in 2015 were white fir ( *Abies concolor* ), ponderosa pine ( *Pinus ponderosa* ), sugar pine ( *Pinus lambertiana* ), and black oak (*Quercus kellogii*), with some incense cedar ( *Calocedrus decurrens* ) and canyon live oak ( *Quercus chrysolepis* ) (Figure 2A). However, this composition was already shifting, as ponderosa pine mortality in particular was quite high toward the end of the exceptional 2012–2016 “hot drought” (Figure 3).

Along with three other research sites within the park, SP was visited bi‐annually by the Moran lab team to measure trees and seedlings and to collect seed trap contents, as well as to check on planted pine seedlings. In September of 2021 the KNP Complex Fire burned through Sequoia and Kings Canyon National Parks. Of our four sites, two were impacted, with SP being the most affected. All understory plants, including all natural and planted seedlings, were killed, as were the vast majority of canopy trees (Figure 2B). The adult sequoias survived, however, as did a line of other trees at the edge of General's Highway (Figure 2C).

Following the fire, we re‐mapped the site, replaced the seed traps, and began counting cone production on remaining trees as part of an NSF‐funded project examining continental‐scale patterns of tree reproduction. Additionally, we began carrying out surveys of natural seedling recruitment and growth along transects of variable length. To carry out these surveys, a reel tape is rolled out such that it passes through an area of reasonably consistent canopy coverage (i.e., full shade, half shade, or no shade along the full transect). The length of the transect is determined partly by the variability in canopy coverage, but also the seedling density and obstacles; longer transects can be used in areas with lower seedling density to allow for more observations, and the transect will be stopped if it runs into a barrier such as a cliff or a rock outcrop. In this case, the transect was 37 m long. All seedlings within 1 m to each side of the tape are measured for their current height and their most recent growth increment, which is defined as the distance from the last bud scar to the tip of the apical meristem.

## 3 Results

Within the 74 m^2^ area of the transect, which ran through an area with zero canopy cover, we observed seedling numbers as shown in Table 1. Giant sequoia seedlings were the most common, with white fir also being abundant. None were first‐year seedlings, indicating that all germinated within the first 2 years after the fire rather than the most recent spring. Notably, except for white fir, none of these species currently exist as adults within 100 m of the transect, suggesting fairly long‐distance dispersal of seed.

**TABLE 1:** Seedling numbers in 74 m^2^ transect.

Giant sequoia	White fir	Ponderosa pine	Incense cedar
65 (0.88/m^2^)	38 (0.51/m^2^)	16 (0.2/m^2^)	2 (0.03/m^2^)

**FIGURE 4:**
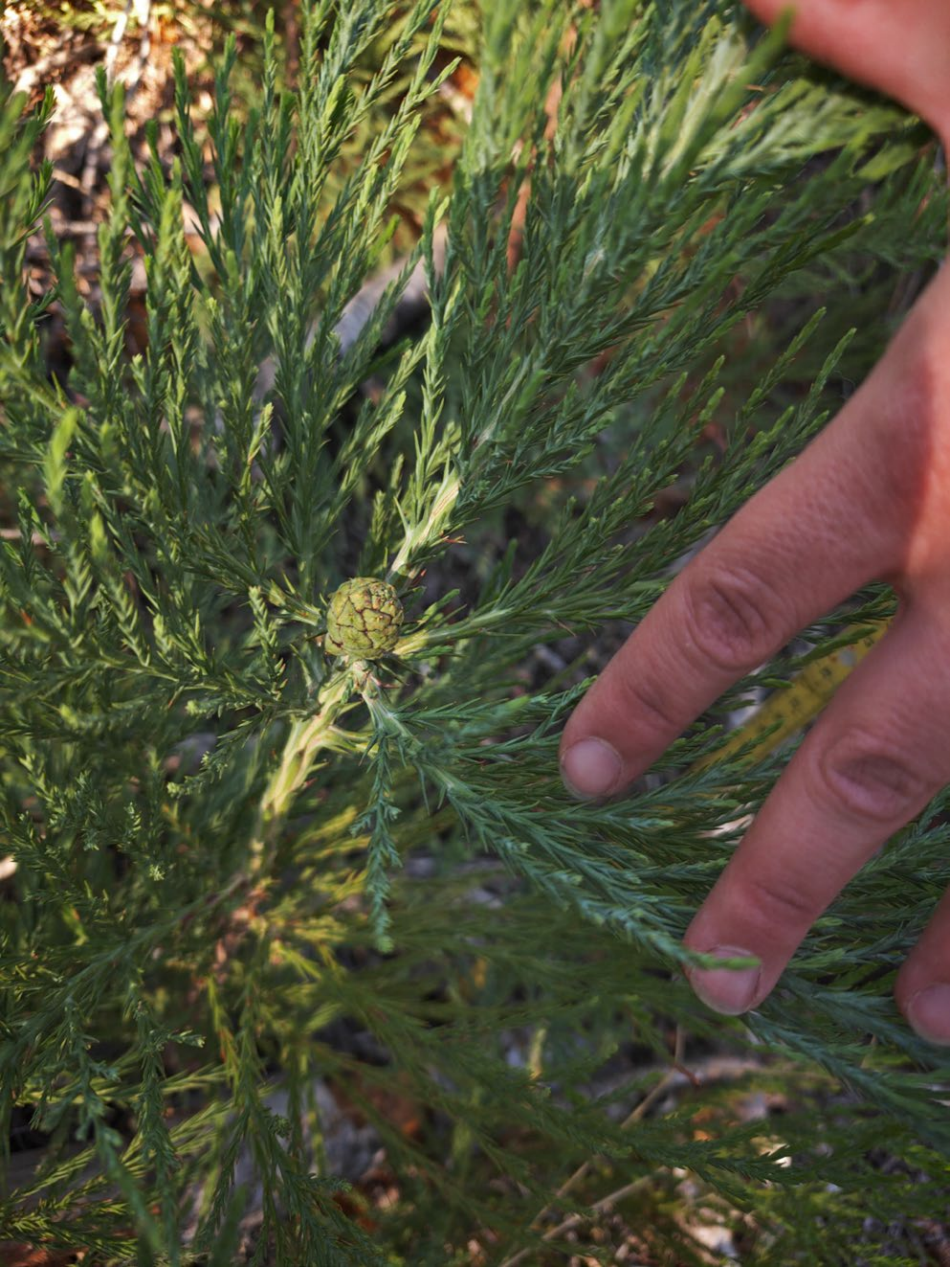
Female cone observed on 3rd year seedling giant sequoia, June 2025. (Photo by Diane Huebner).

Among the sequoia seedlings, one 65 cm tall individual was observed to have developed a single female cone near its apex (Figure 4). Sequoia cones take 2 years to fully develop and are 5–9 cm long when mature (Weatherspoon 1990); given its size, this cone is likely early in its development. The seedling is also not a re‐sprout; surveys showed zero adult or juvenile sequoias inside or within 30 m of the plot borders prior to the fire.

## 4 Discussion

The high recruitment of sequoia seedlings following the fire, and its increase in abundance relative to other species, is consistent with several other studies, both following this severe fire (Hanson, Chi, Khosla, et al. 2024) and others of similar severity (Hanson, Chi, Baker, et al. 2024). In a study focusing on a managed backburn within Tuolumne Grove, for microsites without cones on the ground—like the area of our transect—greater sequoia seedling recruitment was observed for the “heavily burned” category than for less severe burn categories (Lauder et al. 2024). Where severe burn areas are particularly large in extent, the number of seedlings can be reduced relative to less severely burned areas, though numbers often still exceed 1768 seedlings/ha (Soderberg et al. 2024). However, the production of reproductive structures by these young post‐wildfire seedlings has not previously been documented. According to Weatherspoon (1990), cones with fertile seeds have been observed on sequoias as young as 10 years, while large crops rarely are produced before 150–200 years; however, no citation or data is provided for this statement. When Buchholz (1938) examined sequoia branches broken off by an ice storm, trees < 60 years old were only occasionally found to have produced female cones.

Various factors can play a role in individual size at reproductive maturity. For instance, high resource levels—such as elevated nitrogen (Williams et al. 2004) or CO_2_ levels (LaDeau and Clark 2001) ‐ can increase the probability of tree maturation at a given diameter. On the other hand, environmental stress can also favor earlier reproduction in some conifers, possibly because tree growth potential and future reproductive potential are smaller at such sites (Santos‐del‐Blanco et al. 2013). Moreover, as with cases where fishing pressure favored maturation at smaller sizes in fish (Miethe et al. 2010), more intense or frequent disturbance regimes in forests (McDowell et al. 2020) could potentially favor earlier maturation as well. However, reproduction as a 3rd year seedling of < 1 m in height is a rather extreme deviation from the norm, and it is unclear which, if any, of these factors might have contributed.

Reproductive maturation in conifers entails changes to the apical meristems, reflecting changes in gene expression which can even persist for years after mature stems are grafted to juvenile rootstocks; conversely, seedlings grown from tissue cultures of mature meristems may mature more rapidly than seedlings grown from seed (Greenwood 1995). This is what is known as ontogenetic aging, as distinct from the physiological aging that is driven by the size or structural complexity of the plant (Haffner et al. 1991). Mature giant sequoia show changes in carbohydrates (including more cellulose and hemicellulose in the leaf mesophyll), in bud calcium and potassium (though this could be driven by tree size), and changes in peroxidase (linked to rooting potential) (Haffner et al. 1991). Maturation in plants requires a transition in which the cells become able to respond to the internal and external signals that trigger reproductive bud development, a change which seems to be partially governed by microRNAs miR156/miR156 acting on transcription factors SBP/SPL (Poethig 2013). Maturation at a smaller size in plants could be considered a form of paedomorphosis (Box and Glover 2010).

Cone production by seedling giant sequoia is likely extremely rare. Moreover, even if the seeds produced were viable, they would likely not disperse and germinate properly because, being close to the ground, the serotinous cones might fully combust even in the type of lower‐intensity fire with which the species co‐evolved (Lanner 2002) and, even if seed release were triggered, wind dispersal of seeds is more effective at greater heights. However, this extreme case does suggest that a focused investigation of reproductive potential in giant sequoias that are < 60 years old or 30 cm in diameter is needed.

A study of Mountain Ash ( *Eucalyptus regnans* ) provides a useful model for such a study (von Takach Dukai et al. 2018). Mountain ash is, along with giant sequoia and coast redwood ( *Sequoia sempervirens* ), among the tallest trees in the world, and is similarly threatened by decreasing intervals of stand‐replacing fire. As with giant sequoia, their seed capsules are serotinous, requiring exposure to fire to release the seeds. A survey of stands of different ages found a minimum age of reproduction of 11 years—much younger than previously published ages, though flowering can start as soon as age 6–8. However, seed production was still low at age 11, while in 20‐year‐old stands at least 40% of the trees had an average of 208 seed capsules each. The authors note that under conditions of increasing fire frequency, “The substantial variation in fruit capsule numbers between trees within stands suggests that there is potential for strong selection for individuals that reach reproductive viability quickly” (von Takach Dukai et al. 2018).

Given that high‐intensity fires in recent years are estimated to have killed 13%–19% of the total remaining population of giant sequoias (Soderberg et al. 2024), a similar systematic survey of reproductive size or age in sequoia could be important for conservation efforts. Historically, giant sequoia groves burned at low to medium intensity every 15 years on average but, despite their fire adaptations, high intensity fire can kill around 84% of even individuals > 1.2 m at DBH (Shive et al. 2022). If giant sequoia produce viable seeds at smaller ages or sizes than previously thought at non‐negligible frequencies, or if environmental conditions such as high fire severity or high sunlight trigger earlier reproduction, this could help buffer populations against changing fire patterns. While loss of large older trees would still severely impact the cultural value and ecological function of sequoia groves over typical management timespans, smaller trees setting seed might prevent local or range‐wide extinction and, over multi‐century timespans, allow the re‐establishment of “monarch” trees, provided severe fire frequency once again declines. However, this would only be true if potential tradeoffs between early reproduction and growth (Stearns 1989), which much evidence supports for forest trees (Thomas 2011), don't compromise the ability of young sequoia to reach the size at which they are able to withstand mild to moderate fires. Thus, the relationship between growth rate and reproductive status should also be examined.

## Author Contributions

**Emily V. Moran:** conceptualization (lead), funding acquisition (lead), investigation (lead), methodology (lead), resources (lead), supervision (lead), writing – original draft (lead), writing – review and editing (equal). **Diane Huebner:** conceptualization (supporting), investigation (supporting), writing – review and editing (equal).

## Funding

Funding for this research came from NSF, DEB #2211767. Data collection was supported by resources from the Sequoia Field Station (part of UC Natural Reserve System).

## Conflicts of Interest

The authors declare no conflicts of interest.

## Data Availability

Photo of the sequoia seedling and cone, plot location, and seedling density observations are given in the text. Tree number and basal area data used in Figure 3 and the R code to create it are available at https://github.com/emoran5/SEKI‐tree‐figures.
